# Fluralaner systemic treatment of chickens results in mortality in *Triatoma gerstaeckeri*, vector of the agent of Chagas disease

**DOI:** 10.1186/s13071-023-05805-1

**Published:** 2023-06-02

**Authors:** Cassandra Durden, Yuexun Tian, Koyle Knape, Cory Klemashevich, Keri N. Norman, John B. Carey, Sarah A. Hamer, Gabriel L. Hamer

**Affiliations:** 1 Department of Veterinary Integrative Biosciences, Texas A&M University, College Station, USA; 2 Schubot Center for Avian Health, Department of Veterinary Pathobiology, Texas A&M University, College Station, USA; 3 Department of Entomology, Texas A&M University, College Station, USA; 4 Department of Poultry Science, Texas A&M University, College Station, USA; 5 Integrated Metabolomics Analysis Core, Texas A&M University, College Station, USA

**Keywords:** Xenointoxication, Endectocide, Integrated vector control, Triatomine, Poultry

## Abstract

**Background:**

Chagas disease remains a persistent vector-borne neglected tropical disease throughout the Americas and threatens both human and animal health. Diverse control methods have been used to target triatomine vector populations, with household insecticides being the most common. As an alternative to environmental sprays, host-targeted systemic insecticides (or endectocides) allow for application of chemicals to vertebrate hosts, resulting in toxic blood meals for arthropods (xenointoxication). In this study, we evaluated three systemic insecticide products for their ability to kill triatomines.

**Methods:**

Chickens were fed the insecticides orally, following which triatomines were allowed to feed on the treated chickens. The insecticide products tested included: Safe-Guard® Aquasol (fenbendazole), Ivomec® Pour-On (ivermectin) and Bravecto® (fluralaner). *Triatoma gerstaeckeri* nymphs were allowed to feed on insecticide-live birds at 0, 3, 7, 14, 28 and 56 days post-treatment. The survival and feeding status of the *T. gerstaeckeri* insects were recorded and analyzed using Kaplan–Meier curves and logistic regression.

**Results:**

Feeding on fluralaner-treated chickens resulted 50–100% mortality in *T. gerstaeckeri* over the first 14 days post-treatment but not later; in contrast, all insects that fed on fenbendazole- and ivermectin-treated chickens survived. Liquid chromatography tandem mass spectrometry (LC-QQQ) analysis, used to detect the concentration of fluralaner and fenbendazole in chicken plasma, revealed the presence of fluralaner in plasma at 3, 7, and 14 days post-treatment but not later, with the highest concentrations found at 3 and 7 days post-treatment. However, fenbendazole concentration was below the limit of detection at all time points.

**Conclusions:**

Xenointoxication using fluralaner in poultry is a potential new tool for integrated vector control to reduce risk of Chagas disease.

**Graphical Abstract:**

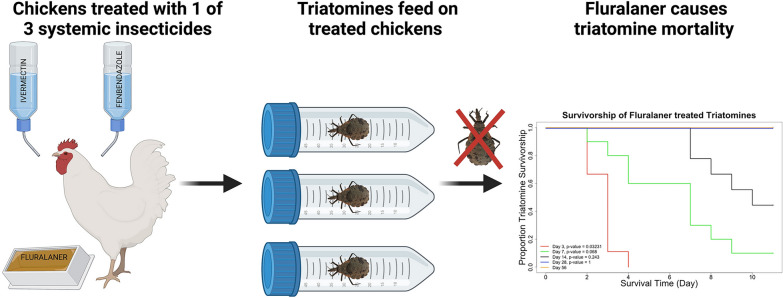

**Supplementary Information:**

The online version contains supplementary material available at 10.1186/s13071-023-05805-1.

## Background

Chagas disease, caused by the flagellate protozoan parasite *Trypanosoma cruzi*, has one of the largest human disease burdens of all vector-borne diseases in the Americas, resulting in an estimated 6,469,283 cases in 2019 [1]. The predominant mode of transmission of *T. cruzi* is stercorarian involving triatomine insects (subfamily Triatominae, Hemiptera: Reduviidae), in which the infectious stages of the parasite are excreted with the insect feces and enter the vertebrate through the biting wound or mucosa [2, 3]. Some animals may also ingest infected bugs, leading to orally-acquired infection [4, 5]. Both *T. cruzi* and triatomines have broad host ranges, and *T. cruzi* infections have been recognized in many species of mammals, including domestic dogs and cats as well as several wildlife species [6–9].

The primary method to reduce Chagas disease is by reducing human contact with infected triatomines, including vector control, household improvements and environmental management [10–12]. In highly endemic regions of the Americas, local ministries of health have relied on the indoor application of residual pyrethroid insecticide to decrease domestic triatomine populations [13]. However, triatomines have demonstrated a capacity for recolonization from sylvatic refugia following the cessation of insecticide treatments [14, 15]; in addition, resistance to pyrethroid insecticides [16] has resulted in control failures [17].

Many species of triatomine bugs (triatomines) play a role in human Chagas disease transmission, with the most notable being *Rhodnius prolixus, Triatoma infestans,* and *Triatoma dimidiata* throughout central and South America, which are commonly found colonizing homes [18]. In the USA, 11 different species of triatomines occur across the 28 southern states. Seven triatomine species found in Texas, with *T. gerstaeckeri* being the most commonly encountered by humans [19]. *Triatoma gerstaeckeri* can be found in both sylvatic and domestic settings in the US states of Texas and New Mexico and in northern Mexico [20], often in association with rodent and armadillo nests, corrals, stables, chicken coops and occasionally human dwellings [19, 21]. Studies estimate up to 55% of adult *T. gerstaeckeri* are infected with *T. cruzi* [21].

Several vertebrate species play a role in *T. cruzi* transmission, either directly as reservoirs or indirectly by providing triatomine blood meals to sustain vector populations. Dogs, cats and chickens are all common blood meal hosts of triatomines in the domicile and peridomicile context and may bring vectors into close contact with humans [23]. In particular, chickens have been found to be positively associated with triatomine abundance [24]. While chickens are not competent hosts for *T. cruzi *[25], they are capable of sustaining vector populations [24]. Chickens are abundant throughout regions of Latin America where Chagas disease is endemic. Smallholder 'family' poultry production is a major source of animal protein throughout Central America, and chickens are the most common domestic fowl found throughout these areas [26, 27].

Treating highly utilized vertebrates with systemic insecticides, resulting in toxic blood meals for triatomines—also called xenointoxication—may provide a solution to reducing Chagas disease in human and animal populations while minimizing non-target effects of insecticide use [28]. Recent studies have shown success in killing triatomines with host-targeted insecticides in dogs using various delivery methods and formulations, including deltamethrin-treated collars, topical solutions and oral systemic insecticides [29–31]. Several of the methods using the active ingredient fluralaner have shown up to 100% mortality of South American triatomine vectors that fed on treated hosts [32, 33]. Ivermectin-containing blood meals from dogs have been shown to be lethal to triatomines, with the most effect within 3 days of dog treatment [34]. However, investigations of xenointoxication of triatomines have not previously considered North American triatomines, and few studies have investigated hosts other than dogs.

To explore the feasability and efficacy of a host-targeted control method to manage triatomine populations, we evaluated survivorship in *T. gerstaeckeri* following consumption of blood meals directly from chickens orally treated with one of the following three active ingredients: fenbendazole, ivermectin and fluralaner. The results from this study will provide insight into the ability to treat chickens with insecticides that kill triatomines.

## Methods

### Study organisms

In this study, a trial was defined as any event during which triatomines are fed on live chickens. Each trial consisted of four chickens, of which three chickens in each trial were treated with one of the three insecticides and one chicken was untreated (control). Each chicken was subjected to feeding by three triatomines (each individually contained). Trials were conducted at six unique time points following treatment of the chickens with insecticide: days 0, 3, 7, 14, 28 and 56 days post-treatment (DPT). Trials on day 0 were conducted twice, and trials on days 3, 7, 14, 28 and 56 were repeated three times. One additional round of trials were conducted at 3, 7, and 14 DPT, but was ended early due to the management practice of the Poultry Facility to apply acaricides to chickens, possibly coming into contact with the birds in our study.

The chickens (*Gallus gallus domesticus;* Hy-line, W-36) used for this study were purchased from the Hy-Line Facility in Bryan, Texas (Hy-Line, West Des Moines, IA, USA). The chickens were all female and hatched on the same date. During the duration of the experiments, the chickens ranged in age from 18 to 38 weeks. The average weight of the chickens used was 1.34 kg. Chickens were housed individually in the Poultry Science Farm at Texas A&M University and provided fresh food (Layer Diet Formation; Texas A&M Poultry Science Center, College Station, TX, USA) and water (300 ml) daily. Individual chickens were only used in a single trial to minimize concern about acquired immunity following exposure to triatomine salivary proteins [35]. Due to molting activity in the triatomine nymphs, three control trials only used one triatomine per chicken. Therefore, a total of 234 triatomines and 80 chickens were used throughout the duration of the study.

*Triatoma gerstaeckeri* were obtained from the colony maintained at Texas A&M University. This colony was housed in a Animal and Plant Health Inspection Service–United States Department of Agriculture (USDA-APHIS) PPQ-approved BSL2 quarantine facility and has been maintained for 6 years at 27–33 °C and 30–60% relative humidity [36, 37]. The insects used in the study are F2-F3 generations removed from wild populations in Texas. Defibrinated rabbit blood (HemoStat Laboratories, Dixon, CA, USA) was provided once per week for feeding using a Hemotek membrane feeder (Hemotek Ltd., Lancashire, UK), but individuals selected for trials were starved for 2 weeks to 1 month [36]. All triatomines were housed in plastic containers lined with filter paper (Whatman plc, Maidstone, UK), with each plastic contained placed within a larger plastic tub containing water-impregnated plaster to maintain humidity [36]. Nymphal triatomines (3rd-5th instar) were used as they take regular blood meals but cannot yet fly.

### Host treatment

Chickens were either given one of three systemic insecticide products (ivermectin, fenbendazole, fluralaner) or no treatment (control chicken) at the same date. The insecticides ivermectin and fenbendazole were delivered in water based on chicken weight, with ivermectin dosed at 0.4 mg/kg body weight (BW) [38] and fenbendazole dosed at 0.005 ml/kg (1mg/kg BW), as indicated on the product label. Chickens treated with these products were given half their normal daily allotment of water (150 ml) to ensure they consumed the full dose of insecticide. These chickens were treated daily for 5 consecutive days, as indicated by previous studies [38]. Chickens were also monitored daily throughout the experiments.

In contrast, chickens treated with fluralaner were given a small oral chewable tablet containing the drug before their food in the morning. Previous studies evaluated fluralaner in a powdered formation and found 0.5 mg/kg to be an optimal dose for chickens (*G. g. domesticus*) to kill poultry red mites (*Dermanyssus gallinae*) [39]. We calculated the necessary weight of the equivalent dose of fluralaner in the dog chewable tablet to be 3.6 mg/kg BW; for the average-sized chicken (1.34 kg), this dose equated to a piece of chew approximately 3 mm in diameter. Dried mealworms were occasionally combined with the chew to improve chickens' willingness to consume, and chickens were watched to ensure complete consumption of this small food item. Fluralaner was given to the chickens twice, 7 days apart [40]. Feathers on the breast of the chicken were trimmed before triatomine feeding to allow better access to skin for feeding. Chickens were placed in a metal tray and bodies were restrained using bandage wrap (Healqu, Jersey City, NJ, USA) to minimize movement and allow the triatomines to obtain a full blood meal. Chicken behavior was recorded and evaluated on a scale of 0 to 2, with 0 being no movement, 1 being average movement and 2 being a significant amount of movement.

### Triatomine feeding

Third- to fifth-instar nymphs of *T. gerstaeckeri* were allowed to feed on the chickens at 0, 3, 7, 14, 28 and 56 DPT. Day 0 post-treatment was defined as the day before treatment began. During each trial, three *T. gerstaeckeri* were fed on each chicken simultaneously. These bugs were housed in individual 50-ml conical tubes covered with a mesh lid. The tubes were then attached to the trimmed area on the chicken using bandage wrap (4 in.; Healqu, LLC, Jersey City, NJ, USA), with the mesh against the skin. This method of attachment allowed bugs to stick their proboscis through the mesh and feed on the chicken. Trials were conducted in a dark environment under red light to simulate the triatomine feeding environment while allowing observation [41]. Triatomines were allowed to feed for 45 min under these conditions. Up to 75 female *Culex quinquefasciatus* mosquitos were also fed on the chickens at the same time as the triatomines to evaluate the effects of these treatments on survivorship. The mosquitos were placed on the chicken’s feet, away from where the triatomines were placed. The mosquito data will be published separately in the future.

After the blood-feeding event, the engorgement level and weight of each *T. gerstaeckeri* were recorded, and the triatomines were then held in a Peltier incubator (ShelLab, Cornelius, OR, USA) at 26.7 °C and 50% relative humidity. Engorgements were given a number (ranging from 0 to 3) to correlate with the size of the blood meal, with 0 indicating unfed and 3 indicating fully fed, similar to methods used by Reithinger et al. [31]. A “fed” triatomine was defined as any individual bud with an engorgement score ≥ 1. The survivorship of individuals was recorded every 24 h for 10 days after the trial date. Individuals that exhibited signs of morbidity, defined as movement impairment with varied progression outcomes, were considered dead in the mortality analysis. All individuals with signs of morbidity died within the 10-day observation period.

### Treatment concentrations in serum

To quantify fluralaner and fenbendazole in the chicken serum, 2.5 ml of whole chicken blood was collected in 3.0-ml BD Vacutainer® blood collection tubes (BD Manufacturing, Glenboro, MB, Canada) and then centrifuged at 10,000 RPM for 20 min, following which 1.0 ml of serum was transferred into microcentrifuge tubes (VWR International, Radnow, PA, USA). Serum samples were stored for approximately 3 months at − 20 °C before testing. Targeted liquid chromatography tandem mass spectrometry (LC-QQQ) analysis was performed on a TSQ Quantiva mass spectrometer (Thermo Fisher Scientific, Waltham, MA, USA) coupled to a binary pump UHPLC (Ultimate3000; Thermo Fisher Scientific). Scan parameters for target ions in fluralaner and fenbendazole are given in Table 1. Chromatographic separation was achieved on a Hypersil Gold 5 µm, 50 mm × 3-mm C18 column (Thermo Fisher Scientific) maintained at 30 °C, using a solvent gradient method. Sample acquisition and data analysis were performed using Trace Finder 3.3 application (Thermo Fisher Scientific). Analysis was performed at the Integrated Metabolomics Analysis Core at Texas A&M University.

**Table 1:** Scan parameters for target ions

Insecticide	Polarity	Precursor (*m/z*)	Product (*m/z*)
Fenbendazole	Positive	300	131.1
Fenbendazole	Positive	300	159
Fenbendazole	Positive	300	268.1
Fluralaner	Negative	554.1	424.1
Fluralaner	Negative	554.1	494.1
Fluralaner	Negative	554.1	534.1

### Statistics

All statistical analyses were conducted using R (version 4.2.2; R Foundation for Statistical Computing, Vienna). *Triatoma gerstaeckeri* survival data were analyzed using Kaplan–Meier survival curve ® package: survival) and subsequently compared using paired log-rank test (R package: survminer). Binary logistic regression was used to evaluate the effects of engorgement on survival, as well as the effects of treatment, DPT, interaction of treatment and DPT and life stage on feeding success ® package: stats), with chicken behavior as a random effect. The receiver operating characteristic (ROC) was used for model goodness-of-fit test (R Package: pROC). Ordered logistic regression was used to analyze the effects of treatment, DPT and life stage on engorgement levels (R package: MASS). Model performance was evaluated using the Hosmer–Lemeshow goodness-of-fit test (R package: generalhoslem).

## Results

### Feeding success

**Fig. 1:**
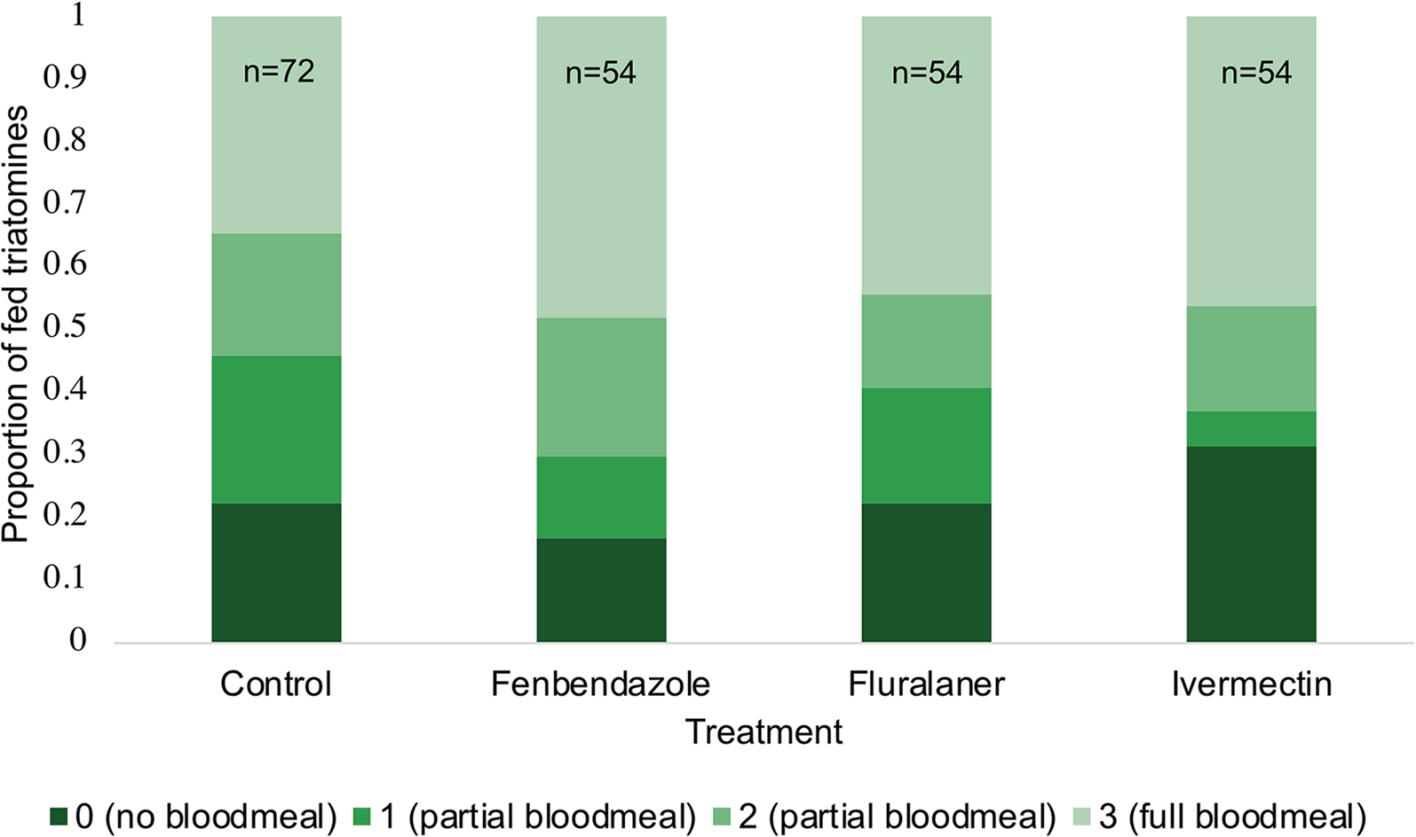
Feeding success and engorgement level of *Triatoma gerstaeckeri* that were fed on treated and control chickens

Feeding success of the *T. gerstaeckeri* ranged from 68.6% to 83.3% under different chicken treatments, with an average of 77.4% (Fig. 1). Of the fed *T. gerstaeckeri*, the majority took a full blood meal (engorgement level 3). Treatment, life stage and DPT did not significantly affect feeding success and engorgement level in our analysis (Table 2). ROC analysis of the logistic regression for variable effects on feeding success resulted in an area under the curve (AUC) value of 0.683, indicating acceptable model fitness (Additional file 1: Figure S1A). Also, product, DPT and life stage were not seen to have an effect on engorgement (or blood meal size) in our analysis. Hosmer–Lemeshow tests conducted on the ordinal logistic regression model resulted in a* P*-value of 0.3986 (*df* = 11, *χ*^2^ = 11.55), indicating acceptable model fitness.

**Table 2:** Logistic regression analysis of potential factors affecting blood feeding success, engorgement, and survivorship

Response variables	Explanatory variables	Levels in model	Odds ratio	95% Confidence interval	*P*-value
Blood feeding success	Product	Control	Reference		
		Fluralaner	1.11	0.31–3.71	0.95
		Ivermectin	0.99	0.34–3.78	0.86
		Fenbendazole	1.04	0.74–13.3	0.15
	DPT		0.65	0.96–1.04	0.74
	Life stage	3rd instar	Reference		
		4th instar	0.30	0.13–2.50	0.57
		5th instar	0.97	0.06–1.15	0.11
	DPT × Product	Fluralaner	0.97	0.96–1.08	0.59
		Ivermectin	10.1	0.92–1.02	0.25
		Fenbendazole	1.02	0.91–1.01	0.18
Engorgement	Product	Control	Reference		
		Fluralaner	1.31	0.68–2.52	0.42
		Ivermectin	1.15	0.59–2.24	0.69
		Fenbendazole	1.45	0.76–2.80	0.27
	DPT		1.00	0.99–1.02	0.85
	Life stage	3rd instar	Reference		
		4th instar	0.43	0.80–4.46	0.14
		5th instar	0.48	0.35–1.94	0.66
Survivorship	Engorgement score^a^	1	Reference		
		2	0.40	0.11–1.28	0.13
		3	0.23	0.08–0.64	0.01*

*DPT* Days post-treatment

*Statistically significant at *P* ≦ 0.05

^a^Engorgements were scored with a number (ranging from 0 to 3) to correlate with the size of the blood meal, with 0 indicating unfed and 3 indicating fully fed

### Survivorship

**Fig. 2:**
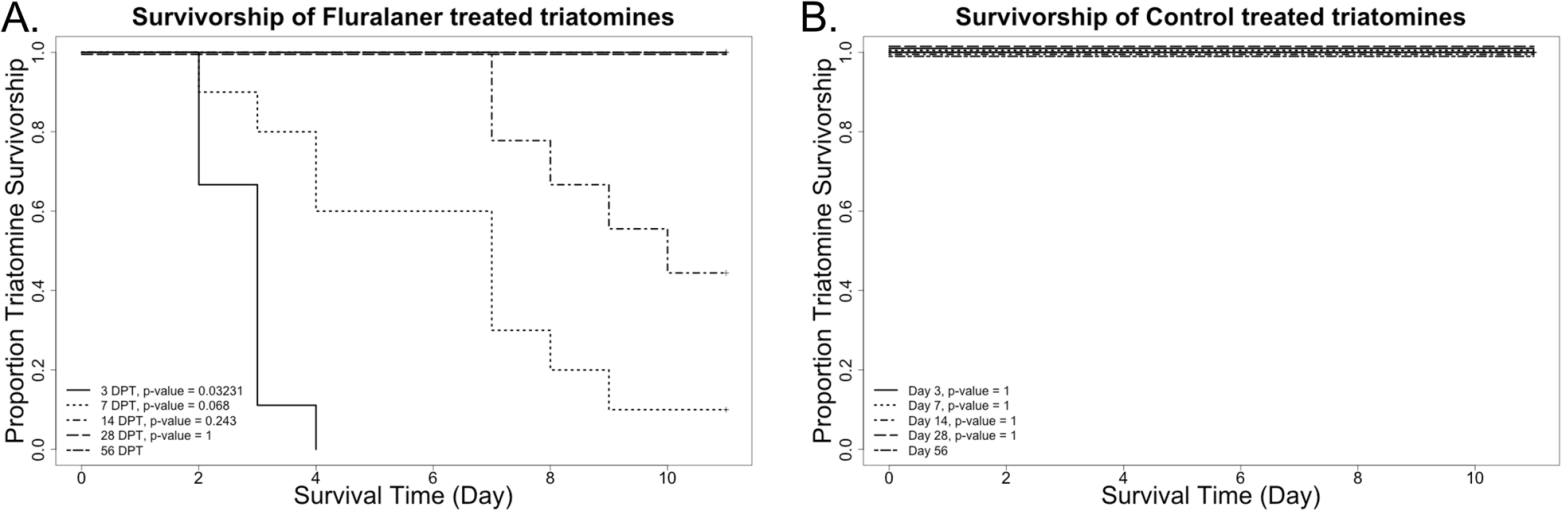
Kaplan-Meyer survivorship curves for triatomines that fed on chickens treated with fluralaner (**a**) and triatomines that fed on untreated control chickens (**b**). The survival curve at 56 DPT was compared to others with an alpha level of 0.05. DPT, Days post treatment

Of the three products, only fluralaner showed any effect on the survivorship of the *T. gerstaeckeri* insects (Fig. 2). At 3 DPT, all *T. gerstaeckeri* that fed on fluralaner-treated chickens died within 4 days of blood feeding. However, the efficacy of fluralaner decreased over time: at 7 and 14 DPT, 90% and 50% of *T. gerstaeckeri* that fed on fluralaner-treated chickens, respectively, died within 10 days, and no mortality was observed in *T. gerstaeckeri* that fed on fluralaner-treated chickens at 28 or 56 DPT. For the Kaplan–Meyer analysis, we defined any insect that lived past the 10-day observation time as living 11 days, indicating that the insects had lived past the observable range. Survivorship of *T. gerstaeckeri* varied by engorgement score, in which insects with an engorgement score of 3 were 77% less likely to die compared to those with an engorgement score of 1 (logistic regression, *P*-value = 0.01, odds ratio [OR] 0.23, 95% confidence interval [CI] 0.08–0.64; Table 2). There was 100% survival for insects that fed on the control-, ivermectin- and fenbendazole-treated chickens at all time points. ROC analysis conducted on this model correlated with an AUC value of 0.659, indicating acceptable model fitness (Additional file 1: Figure S1B).

### Serum concentrations and efficacy of treatment protocol

The chicken serum concentrations of fluralaner had no consistent pattern observed across DPTs and replicates, which ranged from 93.5 ng/ml to below the limit of quantification (2.5 ng/ml) (Table 3). All serum samples were below the lower limit of quantification for fenbendazole (< 5 ng/ml).

**Table 3:** Concentrations of product active ingredients in chicken blood sample

Product	DPT	Repetition^a^	*N*^b^	Concentration (ng/ml)^c^	Engorgement^d^	Survival time (days)^d^
Fluralaner	3	1	2	43.05	2.0	3.5
		2	1	4.45	3.0	3
		3	3	LOQ	3.0	2
	7	1	2	65.9	1.0	7.5
		2	3	2.52	3.0	8.3
		3	3	93.55	1.7	5.3
	14	1	1	24.92	3.0	11
		2	3	LOQ	1.7	10
		3	2	5.40	3.0	11
	28	1	2	LOQ	2.5	11
		2	2	LOQ	2.5	11
		3	3	LOQ	3.0	11
	56	1	3	LOQ	2.7	11
		2	3	LOQ	2.3	11
		3	2	LOQ	2.0	11
Fenbendazole	3	1	3	LOQ	2.0	11
		2	3	LOQ	2.0	11
		3	3	LOQ	3.0	11
	7	1	2	LOQ	2.0	11
		2	2	LOQ	2.5	11
		3	3	LOQ	3.0	11
	14	1	2	LOQ	3.0	11
		2	2	LOQ	2.0	11
		3	3	LOQ	2.0	11
	28	1	0	LOQ	NA^e^	NA^e^
		2	3	LOQ	2.0	11
		3	3	LOQ	2.0	11
	56	1	2	LOQ	3.0	11
		2	3	LOQ	3.0	11
		3	1	LOQ	3.0	11

*DPT* Days post-treatment

^a^Total of 3 repetitions of each trial

^b^Number of triatomines that fed on chicken

^c^LOQ = less than the limit of quantification. Fluralaner: 2.5 ng/ml; fenbendazole: 5 ng/ml

^d^Mean of the fed triatomines

^e^Not applicable; no triatomines took a blood meal from this chicken

## Discussion

We report here for the first time that blood-feeding on fluralaner-treated chickens resulted in subsequent mortality in triatomines. These results further emphasize the potential of fluralaner as an effective drug for xenointoxication, as well as provide a proof-of-concept for the addition of poultry to host-targeted interventions for Chagas disease management. Fluralaner is a member of the isoxazoline drug class and has been used in products to treat ectoparasites of various animals, including dogs, cats and, more recently, chickens [39, 40]. In the USA and other locations, fluralaner is the active ingredient in an oral chewable medication given to dogs to treat fleas and ticks under the name Bravecto® (Merck Animal Health USA, Rahway, NJ, USA).

Fluralaner treatment of chickens resulted in total *T. gerstaeckeri* mortality through to 14 DPT. This results matched the measurements of fluralaner concentration in the chicken serum, which showed detectable levels at 3, 7 and 14 DPT but levels below the detectable limit at 28 and 56 DPT. Our results corroborate those of similar studies performed with chickens treated with fluralaner for red mite treatment, in which the results suggested that the product shows the highest amount of efficacy within the first 2 weeks of treatment [42]. Other recent studies have found similar success in evaluating the use of fluralaner for control of the common bed bug *Cimex lectularius* in poultry farms [43]. The concentration of fluralaner in chicken plasma at different DPT was inconsistent among individual chickens, possibly explained by variation in the oral consumption of the chewable product, although chickens were observed for complete consumption of the food granules containing the insecticide, or heterogeneity of the fluralaner concentration in the chewable tablet.

Fluralaner has previously been evaluated as a potential control tool for Chagas disease through xenointoxication of domestic dogs, with the authors reported up to 100% mortality for 7 months in *Triatoma brasiliensis* [30]. Field trials in Argentina further revealed that treating dogs with fluralaner resulted in reduced *T. infestans* populations [44]. Our study reveals the potential for chickens to be added to host-targeted strategies for Chagas disease management, and future research should evaluate the effects of fluralaner on different triatomine species, as well as on triatomines that are resistant to other insecticides.

Fenbendazole is a broad-spectrum antihelminth in the benzimidazole drug class and has been evaluated in many animals, including cattle, dogs, chickens and others [45]. Safe-Guard® AquaSol (Merck Animal Health USA), a product that contains fenbendazole as the active ingredient, is commercially available as an additive to chicken drinking water. While fenbendazole is not known to have activity against ectoparasites [46], it is one of the few antiparasitic drugs labeled for use in poultry in the USA. We observed no mortality in *T. gerstaeckeri* feeding on fendbendazole-treated chickens at 3 DPT and beyond. All plasma concentrations of fenbendazole were less than 5 ng/ml, indicating that the minimum concentration to cause mortality in *T. gerstaeckeri* is higher than 5 ng/ml.

Ivermectin is an endectocide belonging to the macrocyclic lactone class that has been used to treat intestinal parasites of dogs, chickens, cats and other animals [47]. It is the active ingredient of Ivomec® Pour-On (Boehringer Ingelheim, Biberach, Germany), which is a commercial product available as a pour-on solution to treat intestinal parasites in cattle, as well as used off label as a food additive and a water additive [38]. It has recently been assessed as a treatment for ectoparasites, including bed bugs and mosquitos [38, 43, 48]. Pharmacokinetic studies of ivermectin found that it reaches maximum concentration immediately after treatment and may reach the limit of quantification within 24 h [49]. Similarly, Nyguyen et al. found that the levels of ivermectin in chicken serum dropped quickly, with levels in the serum peaking at 24 h post-treatment and continuing to have a significant effect on mosquito mortality only until 3 DPT; by 5 DTP, levels had reached below the lethal concentration, resulting in 50% mortality (LC_50_) [38]. Experiments done in triatomines found that while 83.3% of triatomines which ingested ivermectin-containing blood meals of dogs died within 24 h, mortality was reduced to only 13% by day 6 [34]. Although ivermectin can cause high arthropod mortality, its effect appears to be restricted to the first few days of treatment, likely due to factors such as rapid detoxification, clearance from the blood and high metabolic rate [43]. Given the interest in our study to identify single-dose interventions rather than continuous use interventions, the design of our study did not capture the acute timeframe over which ivermectin is expected to kill blood-feeding vectors. These results ultimately suggest that ivermectin may not be effective for long-term treatment of ectoparasites [43], unless the dose is given consistently.

Not all *T. gerstaeckeri* that were applied to a chicken engaged in blood-feeding. To consider the role of chicken movement in feeding success, we included chicken behavior as a random effect in the logistic regression. We found that DPT, life stage and treatment did not significantly affect the feeding success of the triatomines. Previous studies have shown that in some cases, insects, such as sand flies, may be repelled by an insecticide-treated host [50] but could subsequently divert the vector to nearby untreated hosts, such as humans. This could influence parasite transmission dynamics by encouraging vectors to avoid feeding on treated dogs and instead feed on humans. However, we did not find a difference in feeding success across treatments and, therefore, our findings did not suggest any repellent effect of *T. gerstaeckeri* in any of the products evaluated.

While we expected to find that a higher engorgement would correlate to a higher percentage of mortality, we found that *T. gerstaeckeri* with an engorgement value of 3 were less likely to die than insects with an engorgement value of 1 (Table 2). Although a larger blood meal may be assumed to contain a higher dose of insecticide, the concentrations of insecticide did not show a consistent pattern in the plasma of treated chickens in our study (Table 3).

In combination with xenointoxication of other common triatomine hosts, such as dogs and cats, treating chickens with systemic insecticides may allow triatomines to be controlled in the domicile and peridomicile environment. Fluralaner products can be delivered to chickens as oral treats, as demonstrated in this study, or as a liquid additive to water, as done with Exzolt™ (Merck Animal Health USA), the poultry product for poultry mite control. Exzolt™ is not currently approved by the U.S. Food and Drug Agency although this product should be evaluated for the control of additional blood-feeding arthropods, such as triatomines. Xenointoxication may be especially effective when used in combination with other control methods, including housing modifications and insecticide spraying.

## Conclusions

The results of this study demonstrate that fluralaner induces mortality of *T. gerstaeckeri* after these insects take a blood meal from fluralaner-treated chickens. Xenointoxication of chickens may be used as a potential method to control vectorial transmission of *T. cruzi*, the etiological agent of Chagas disease.

## Supplementary Information

**Additional file 1: Figure S1.** ROC analysis of the logistic regression models for: factors affecting *Triatoma gerstaeckeri* feeding success (**A**) and factors affecting *Triatoma gerstaeckeri* survivorship (**B**).

## Data Availability

Data supporting the conclusions of the present study are included within the article. Data used and/or analyzed during this study are available from the corresponding author upon reasonable request.

## Abbreviation

DPT: Days post-treatment
