# Correction: Fluralaner systemic treatment of chickens results in mortality in *Triatoma gerstaeckeri*, vector of the agent of Chagas disease

**DOI:** 10.1186/s13071-025-06667-5

**Published:** 2025-01-21

**Authors:** Cassandra Durden, Yuexun Tian, Koyle Knape, Cory Klemashevich, Keri N. Norman, John B. Carey, Sarah A. Hamer, Gabriel L. Hamer

**Affiliations:** 1 Department of Veterinary Integrative Biosciences, Texas A&M University, College Station, USA; 2 Schubot Center for Avian Health, Department of Veterinary Pathobiology, Texas A&M University, College Station, USA; 3 Department of Entomology, Texas A&M University, College Station, USA; 4 Department of Poultry Science, Texas A&M University, College Station, USA; 5 Integrated Metabolomics Analysis Core, Texas A&M University, College Station, USA

**Correction: Parasites & Vectors (2023) 16:178** 10.1186/s13071-023-05805-1

Following publication of the original article, the authors brought two errors to the attention of the journal: in the Methods subsection 'Host treatment', in place of (the correct) 'ivermectin dosed at 0.4 mg/kg body weight (BW) [38] and fenbendazole dosed at 0.005 ml/kg (1mg/kg BW)', 'ivermectin dosed at 0.4 ml/kg body weight (BW) [38] and fenbendazole dosed at 0.5 ml/kg BW' had been erroneously written; in the references, reference 38 was incorrect. These errors have since been [1] corrected in the published article. The authors thank you for reading this erratum and apologize for any inconvenience caused.
