# Supplementary material for: Fluralaner systemic treatment of chickens results in mortality in Triatoma gerstaeckeri, vector of the agent of Chagas disease
Source: Parasit Vectors. 2023 Jun 2;16:178. doi: 10.1186/s13071-023-05805-1 (PMC10236763; doi:10.1186/s13071-023-05805-1)
Supplement: Supplementary file 1 — Additional file 1: Figure S1. ROC analysis of the logistic regression models for: factors affecting Triatoma gerstaeckeri feeding success (A) and factors affecting Triatoma gerstaeckeri survivorship (B). [file 13071_2023_5805_MOESM1_ESM.docx]

**Additional File 1**


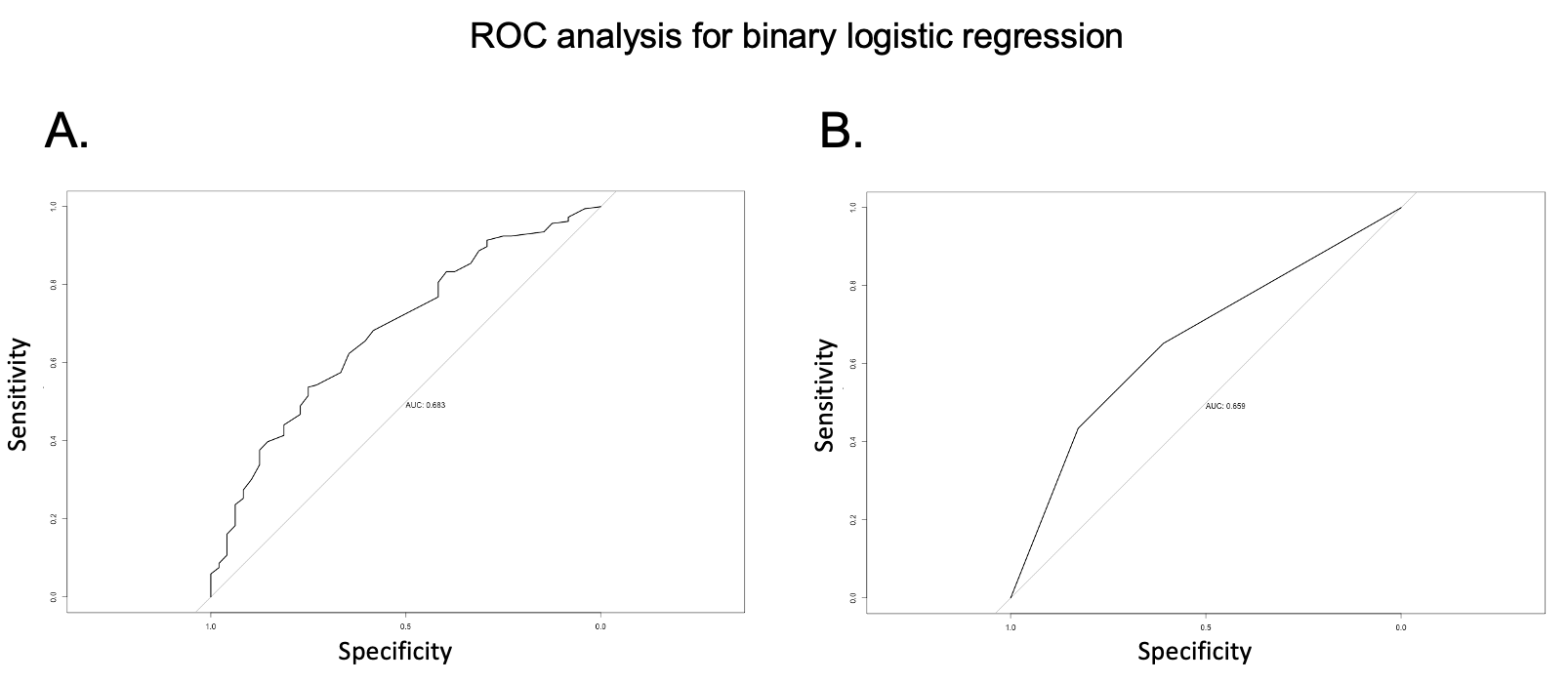


**Figure S1**: ROC analysis of the logistic regression models for A. factors affecting *Triatoma gerstaeckeri* feeding success; B. factors affecting *Triatoma gerstaeckeri* survivorship.
